# Incidence and clinical significance of biliary and pancreatic duct variations on magnetic resonance cholangiopancreatography

**DOI:** 10.1007/s00261-026-05405-4

**Published:** 2026-03-09

**Authors:** Merve Ozkaya, Irfan Atik, Samet Ozkaya, Bulent Yildiz, Mehmet Atalar

**Affiliations:** 1 Department of Radiology, Sivas Cumhuriyet University Faculty of Medicine, Sivas, Turkey; 2 Department of Radiology, Sivas Numune Hospital, Sivas, Turkey

**Keywords:** Magnetic resonance cholangiopancreatography, Biliary tract, Pancreatic ducts, Variation

## Abstract

**Purpose:**

This study aimed to determine the incidence of anatomical variations of the biliary and pancreatic ducts using Magnetic Resonance Cholangiopancreatography (MRCP) and to evaluate their associations with inflammatory and malignant conditions.

**Methods:**

In this retrospective study, MRCP examinations performed between April 2022 and December 2023 were reviewed through the Picture Archiving and Communication System (PACS) regardless of clinical data. Biliary and pancreatic duct variations; the presence of gallbladder or biliary sludge/stones; findings of acute cholecystitis or pancreatitis; and masses or cysts in the pancreas and biliary tract were assessed. Categorical variables were analyzed using the Chi-square or Fisher’s exact test, with *p* < 0.05 considered statistically significant.

**Results:**

Of the 973 patients, 560 (58%) were female and 413 (42%) were male, with a mean age of 60.47 ± 17.33 years. Biliary tract variations were identified in 522 patients (53.6%), most frequently type II (27.78%), type D (26.25%), and type IIIa (15.52%). Pancreatic duct variations were found in 26 patients (2.67%), predominantly pancreas divisum (53.85%), pancreatobiliary junction anomaly (34.62%), and ansa pancreatica (11.54%). Gallstones were present in 474 patients (48.7%), masses in 93 (9.55%), acute cholecystitis in 177 (17.9%), and acute pancreatitis in 78 (8%). Biliary tract variation was significantly associated with gallstones (*p* < 0.05), while no significant associations were observed for other conditions or for pancreatic duct variations (*p* > 0.05).

**Conclusion:**

MRCP effectively identifies biliary and pancreatic ductal variations, which have important clinical implications. Accurate recognition of these variations may help prevent complications during diagnostic and surgical procedures and improve treatment planning.

## Introduction

The hepatic ductal system consists of the gallbladder, where bile secretion is stored, and the intrahepatic and extrahepatic ducts that facilitate bile flow. While the gallbladder stores and concentrates bile, the biliary tracts are responsible for its transport. These anatomical structures are closely related to the surrounding tissues and may exhibit various anatomical variations [1].

The intrahepatic bile ducts exhibit a distribution parallel to the segmental anatomy of the liver. The convergence of the segmental ducts forms the right and left hepatic ducts, which then join to create the common hepatic duct. Distally, the union of the common hepatic duct with the cystic duct gives rise to the common bile duct. The common bile duct subsequently merges with the pancreatic duct and opens into the second portion of the duodenum at the sphincter of Oddi [2, 3].

The biliary system may exhibit a variety of developmental variations at both intrahepatic and extrahepatic levels [3]. Variations of the cystic and hepatic ducts are particularly important in clinical practice, especially during surgical procedures such as laparoscopic cholecystectomy. These variations may predispose patients to complications, including gallstones, pancreatitis, cholangitis, and biliary malignancies, thereby increasing morbidity. With the growing frequency of liver resection and transplantation, accurate understanding of the biliary anatomy and its potential variations has become even more critical [4].

Anatomical structures and pathologies of the pancreatic and biliary systems can be evaluated using magnetic resonance cholangiopancreatography (MRCP), a noninvasive technique. MRCP employs heavily T2-weighted sequences, allowing for rapid, reliable, and complication-free visualization of the biliary and pancreatic ducts without the need for contrast agents [5].

This study aims to determine the incidence of biliary and pancreatic duct variations using MRCP in a large, unselected clinical population and to explore their associations with common inflammatory and malignant hepatopancreatobiliary conditions, providing a clearer understanding of their potential clinical implications.

## Materials and methods

This study was designed retrospectively and conducted after obtaining approval from the Non-Interventional Clinical Research Ethics Committee of the University (dated 21.03.2024, approval number 2024-03/30). All procedures were performed in accordance with the principles of the Declaration of Helsinki.

Patients who underwent MRCP for any indication between April 2022 and December 2023 were retrospectively reviewed using the Picture Archiving and Communication System (PACS). Patients with a history of cholecystectomy or other hepatobiliary or pancreatic surgery, those in whom mass lesions could distort the anatomy of the biliary or pancreatic ducts, individuals with significant imaging artifacts that impeded evaluation, and those under 18 years of age were excluded from the study. MRCP examinations were primarily requested for the evaluation of suspected biliary obstruction, hepatobiliary enzyme abnormalities, pancreatobiliary pain, suspected choledocholithiasis, and pancreatic or biliary pathology identified on prior imaging.

**Fig. 1:**
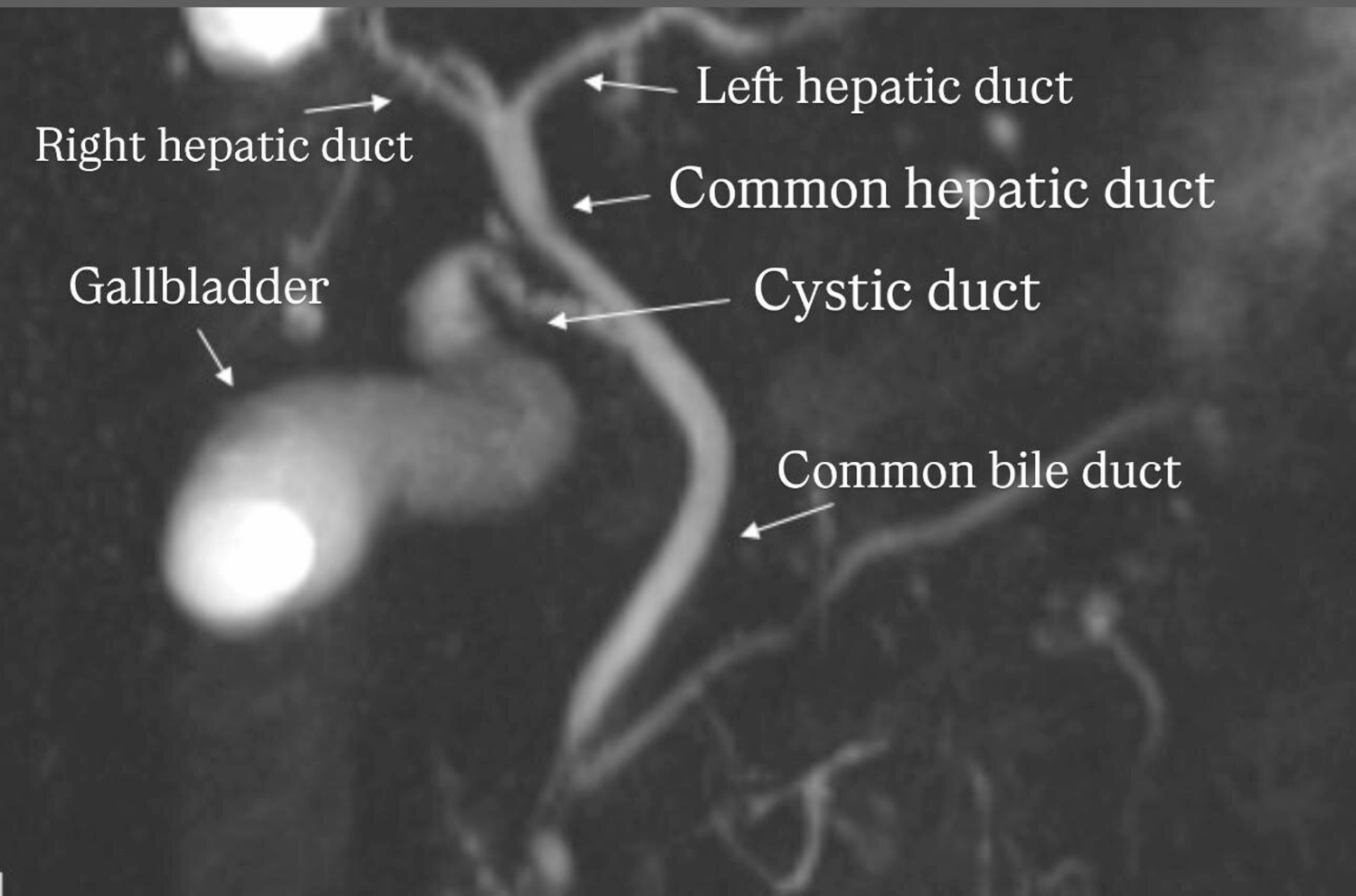
Normal MRCP anatomy of the biliary tree demonstrating the right and left hepatic ducts, common hepatic duct, cystic duct, and common bile duct

Radiological evaluations were performed using a standardized MRCP protocol on a 1.5 Tesla MRI system (Magnetom Aera, Siemens, Erlangen, Germany). Images were obtained using the following sequences: T2-TRUFI coronal (TR: 3.69 ms; TE: 1.85 ms; slice thickness: 6 mm; FOV: 380 mm; matrix: 256 × 256), T2-HASTE axial (TR: 1000 ms; TE: 95 ms; slice thickness: 5 mm; FOV: 380 mm; matrix: 320 × 320), T2-HASTE coronal (TR: 1200 ms; TE: 91 ms; slice thickness: 5 mm; FOV: 380 mm; matrix: 256 × 256), and T2-SPACE coronal p2-trig (TR: 2500 ms; TE: 701 ms; slice thickness: 1.1 mm; FOV: 380 mm; matrix: 384 × 384). No oral or intravenous contrast agent was administered during imaging. Figure 1 shows a representative MRCP image of the normal biliary tree anatomy.

All images were independently evaluated through the Picture Archiving and Communication System (PACS) without knowledge of the patients’ clinical information. In all cases, biliary and pancreatic duct variations were assessed; additionally, the presence of gallbladder and biliary stones, as well as accompanying inflammatory or malignant pathologies, was evaluated. A total of 1,050 MRCP examinations were initially reviewed. Of these, 77 patients were excluded due to a history of cholecystectomy or other hepatobiliary or pancreatic surgery, mass lesions distorting ductal anatomy, significant imaging artifacts, or age under 18 years, resulting in a final study population of 973 patients. All MRCP images were independently evaluated by two radiologists with 9 and 4 years of experience in abdominal imaging, both blinded to clinical data. Interobserver agreement for the identification of biliary and pancreatic duct variations was assessed using the intraclass correlation coefficient (ICC). Discrepant cases were resolved by consensus.

MRCP images of the 973 patients were examined for biliary tract variations, including branching patterns of the right and left hepatic ducts, the configuration of cystic duct insertion into the common bile duct, and potential vascular compression of the common hepatic duct. Pancreatic duct variations were evaluated for the presence of pancreas divisum, pancreaticobiliary junction anomalies, and ansa pancreatica.

**Fig. 2:**
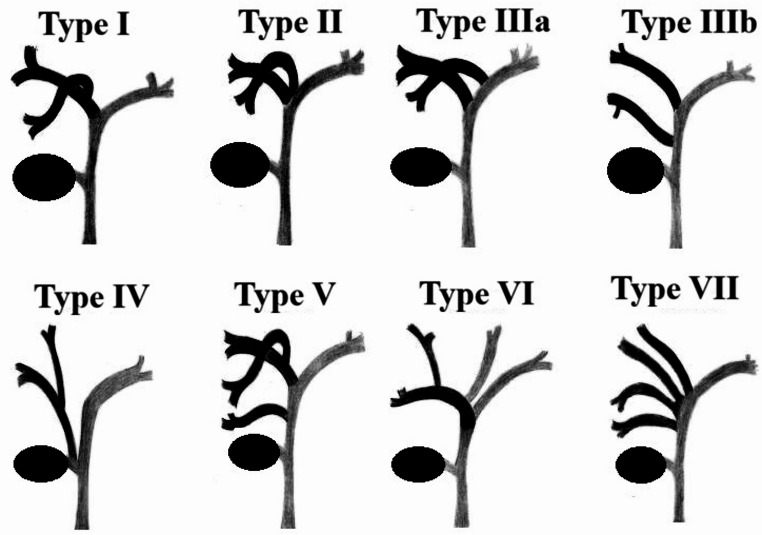
Diagrammatic representation of anatomical variations in the branching pattern of the right hepatic duct, adapted from Jeswani et al. [7]

Branching patterns of the right hepatic duct were classified into seven types (I–VII) based on the branching of the anterior and posterior segmental ducts [6] (Fig. 2).

Type I: Typical pattern in which the right posterior segmental duct (RPSD) joins the right anterior segmental duct (RASD).

Type II: Trifurcation pattern, characterized by the simultaneous drainage of the RPSD, RASD, and left hepatic duct (LHD).

Type III: Anomalous drainage of the RPSD into either the LHD (Type IIIa) or the common hepatic duct (CHD) (Type IIIb).

Type IV: Aberrant drainage of the right hepatic duct (RHD) into the cystic duct.

Type V: Presence of an accessory right hepatic duct.

Type VI: Segment II and III ducts draining into the RHD or CHD.

Type VII: Other unclassified or complex variations.

**Fig. 3:**
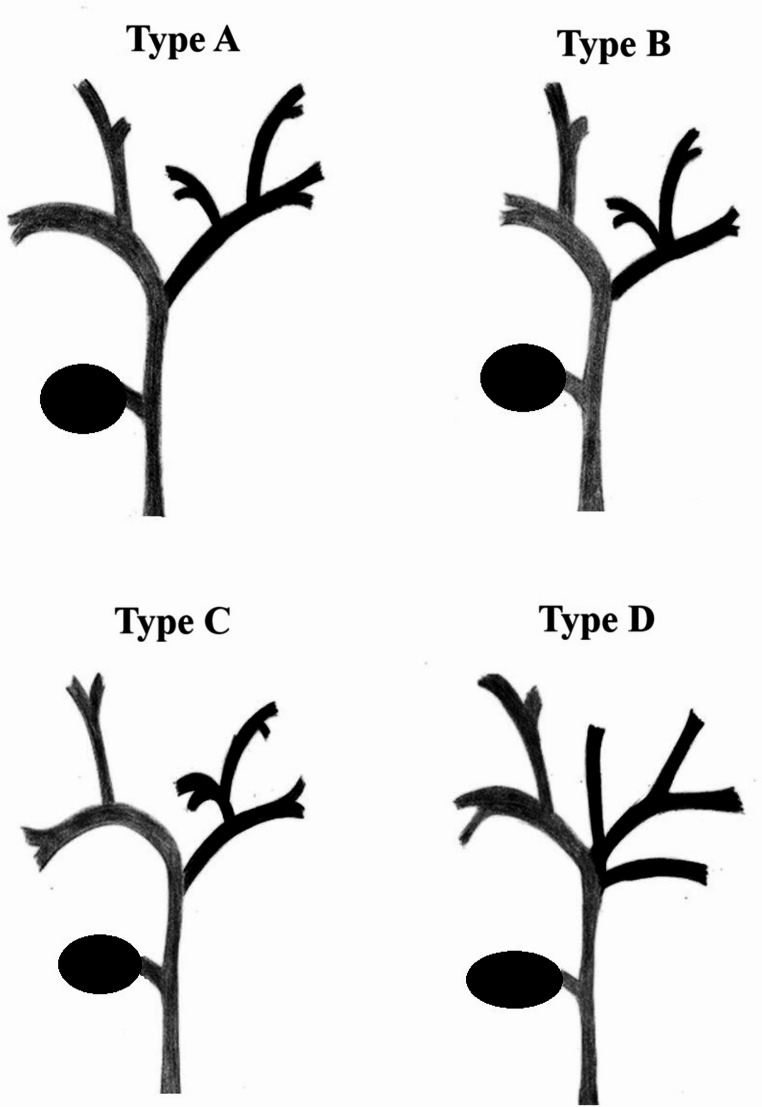
Diagrammatic representation of anatomical variations in the branching pattern of the left hepatic duct, adapted from Jeswani et al. [7]

Branching patterns of the left hepatic duct were classified into four types based on the branching of the segment II, III, and IV ducts [6] (Fig. 3).

Type A: Typical pattern in which a common trunk of segments II and III joins the segment IV duct.

Type B: Trifurcation pattern involving simultaneous drainage of the segment II, III, and IV ducts.

Type C: Drainage of the segment II duct into the common trunk of segments III and IV.

Type D: Other unclassified or complex variations.

**Fig. 4:**
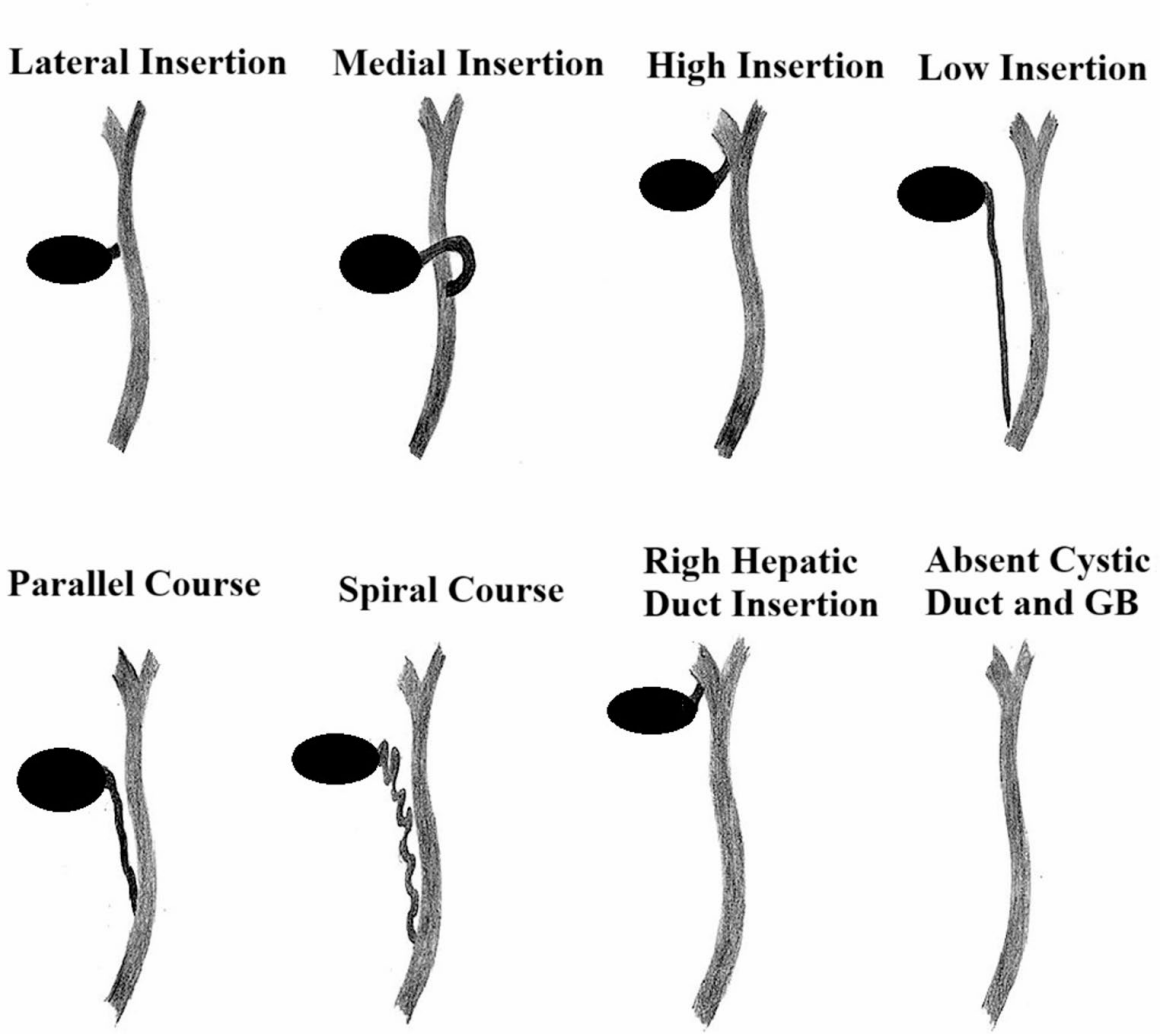
Diagrammatic representation of anatomical variations of the cystic duct, adapted from Jeswani et al. [7]

Cystic duct variations were classified based on their size, course, and configuration of insertion into the common hepatic duct (Fig. 4).

**Fig. 5:**
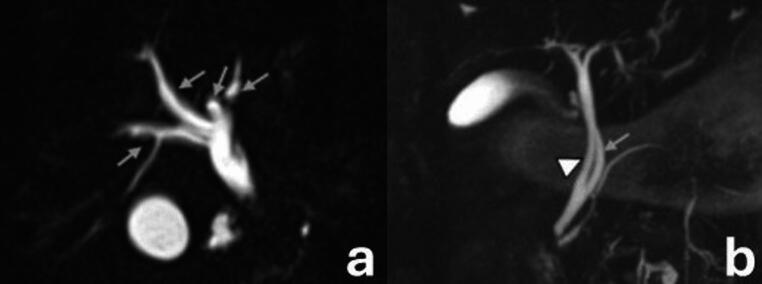
MRCP images demonstrating biliary tract variations: (**a**) Type D biliary tract variation, **b** medial insertion of the cystic duct

Figure 5 presents representative MRCP images of biliary tract variations, illustrating a Type D biliary tract variation (a) and medial insertion of the cystic duct (b).

**Fig. 6:**
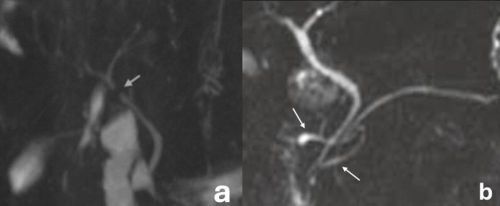
MRCP images showing (**a**) vascular compression of the common hepatic duct (arrow) and (**b**) pancreas divisum, demonstrating absence of communication between the dorsal and ventral pancreatic ducts (arrows)

Compression of the common hepatic duct by the right hepatic artery, crossing anteriorly and appearing as a signal-void area resembling an intraluminal filling defect, was interpreted as vascular compression of the common hepatic duct (Fig. 6) [8].

A pancreaticobiliary junction anomaly was evaluated based on the formation of a common channel ≥ 15 mm in length due to the union of the common bile duct (CBD) and the pancreatic duct in the proximal duodenum [9]. Pancreas divisum is defined as an anatomical variation resulting from the failure of fusion between the dorsal and ventral pancreatic ducts [10]. In this condition, the CBD drains into the major papilla via the ventral pancreatic duct, while the dorsal pancreatic duct drains into the minor papilla. In our study, the main pancreatic duct, draining the tail and body of the pancreas, was observed to pass anterior to the CBD and independently open into the duodenum at a more proximal location. This finding was classified as pancreas divisum (Fig. 6).

Ansa pancreatica is characterized by focal atrophy of an accessory duct and the presence of an additional curved duct connecting the main and accessory ducts in place of the atrophied duct. Ansa pancreatica is considered a predisposing factor for recurrent pancreatitis. During pancreatic duct development, a curved accessory duct forms to replace the atrophied accessory duct. This accessory duct originates from the main pancreatic duct, follows a hooked course anterior to the main duct, and terminates at or near the minor papilla [11] (Table 1).

**Table 1:** Classification and definitions of pancreatic duct anomalies

Pancreatic duct anomaly	Imaging characteristics	Key reference
Pancreaticobiliary junction anomaly	Common channel ≥ 15 mm formed by union of the CBD and pancreatic duct	[9]
Pancreas divisum	Non-fusion of dorsal and ventral pancreatic ducts with separate drainage into the major and minor papillae	[10]
Ansa pancreatica	Curved accessory duct connecting the main pancreatic duct to the minor papilla, replacing an atrophied accessory duct	[11]

Patients were additionally evaluated for the presence of gallstones or biliary sludge, radiological findings of acute cholecystitis and acute pancreatitis, as well as space-occupying lesions in the gallbladder, biliary tracts, or pancreas, and pancreatic cysts.

Oval or round hypointense lesions observed in the gallbladder and biliary tracts were interpreted as stones, whereas layered hypointense signals were considered biliary sludge. Cases with a hydropic gallbladder, increased wall thickness, and signal intensity changes in the surrounding fatty planes consistent with inflammation were classified radiologically as acute cholecystitis. Cases with an enlarged pancreatic caliber and the presence of fluid or increased signal intensity in the peripancreatic and pararenal regions were evaluated as acute pancreatitis. Space-occupying lesions in the gallbladder, biliary tracts, or pancreas with irregular margins were considered masses, whereas well-circumscribed, hyperintense lesions on T2-weighted sequences in the pancreas were interpreted as cysts.

Statistical analyses were performed using SPSS version 23.0. Categorical variables were presented as n (%), and continuous variables as mean ± standard deviation (SD) or median (minimum–maximum). Nominal data were additionally presented in cross-tabulations. Interobserver agreement was evaluated using the intraclass correlation coefficient (ICC). ICC values < 0.50 were considered poor agreement, 0.50–0.75 moderate agreement, 0.75–0.90 good agreement, and > 0.90 excellent agreement. Differences between groups were assessed using the Chi-square test for categorical data in large samples, and Fisher’s exact test for small samples or low cell frequencies. Multivariable logistic regression was performed to adjust for potential confounding effects of age and sex when assessing associations between biliary tract variations and gallstone disease. A p-value of < 0.05 was considered statistically significant.

## Results

Interobserver agreement between the two radiologists for the assessment of biliary and pancreatic duct variations was excellent (ICC = 0.91). Among the 973 patients included in the study, biliary tract variations were observed in 522 cases (53.6%). The most frequent biliary variation was Type II (27.78%), followed by Type D (26.25%) and Type IIIa (15.52%). Certain biliary tract variations were not observed in our study cohort. Pancreatic duct variations were identified in 26 cases (2.67%), of which 14 (53.85%) had pancreas divisum, nine (34.62%) had a pancreaticobiliary junction anomaly, and three (11.54%) exhibited an ansa pancreatica variation (Table 2).

**Table 2:** Distribution of biliary tracts and pancreatic duct variations

				*n*		%	
Biliary tracts variations				522			
		Type II		145		(27.78)	
		Type D		137		(26.25)	
		Type IIIa		81		(15.52)	
		Distal medial insertion of the cystic duct		51		(9.77)	
		Medial insertion of the cystic duct		46		(8.81)	
		Type IIIb		29		(5.56)	
		High insertion of the cystic duct		16		(3.07)	
		Low insertion of the cystic duct		9		(1.72)	
		Type IV		4		(0.77)	
		Vascular compression of the common hepatic duct		3		(0.57)	
		Type V		1		(0.19)	
**Pancreatic Duct Variations**				**26**			
		Pancreas divisum		14		(53.85)	
		Pancreaticobiliary junction anomaly		9		(34.62)	
		Ansa pancreatica		3		(11.54)	

Among the 973 patients, gallstones were detected in the gallbladder or biliary tracts in 474 cases (48.7%), and biliary sludge was observed in the gallbladder in 59 cases (6.1%). Mass lesions in the biliary system or pancreas were identified in 93 patients (9.55%), of which 54 (58.06%) were classified as pancreatic cysts. Additionally, acute cholecystitis was observed in 177 cases (17.9%) and acute pancreatitis in 78 cases (8%).

The study cohort was divided into two groups: patients with and without biliary tract variations. No significant difference was observed between the groups regarding gender distribution (*p* = 0.883). The presence of acute cholecystitis was also similar between the groups and did not reach statistical significance (*p* = 0.378). The frequency of gallstones in the gallbladder and biliary tracts differed significantly between the groups (*p* = 0.001). The presence of mass lesions in the gallbladder or biliary tracts was not significantly associated with biliary tract variations (*p* = 0.069) (Table 3). No statistically significant difference was found between biliary variation types and the presence of gallstones in the gallbladder (*p* > 0.05). When individual biliary tract variation subtypes were analyzed, no specific variation type showed a statistically significant association with bile duct or gallbladder stone presence (*p* > 0.05). The observed association was related to the presence of biliary tract variation as a whole rather than to a particular anatomical subtype. After adjusting for age and sex in a multivariable logistic regression model, the association between biliary tract variations and gallstone disease remained statistically significant (OR 1.75, 95% CI 1.12–2.74, *p* < 0.05), suggesting that this relationship is not solely explained by these demographic factors.

**Table 3:** Comparison of demographic and clinical findings according to biliary tract variations

		Biliary Tract Variation		
		No	Yes	*p*
Gender	Female	278 (57.3%)	282 (57.8%)	0.883*
n (%)	Male	207 (42.7%)	206 (42.2%)	
Acute cholecystitis	No	393 (81.0%)	406 (83.2%)	0.378*
n (%)	Yes	92 (19.0%)	82 (16.8%)	
Gallstone	No	246 (50.7%)	194 (39.8%)	0.001*
n (%)	Yes	239 (49.3%)	294 (60.2%)	
Bile mass	No	484 (99.8%)	481 (98.6%)	0.069**
	Yes	1 (0.2%)	7 (1.4%)	

*Chi-square test

**Fisher’s exact test; not statistically significant (*p* > 0.05)

Patients with and without pancreatic duct variations were compared in terms of gender, the presence of acute pancreatitis, and the presence of pancreatic cysts or masses. No statistically significant differences were observed between the groups regarding gender distribution (*p* = 0.413), acute pancreatitis (*p* = 0.161), or pancreatic cysts/masses (*p* = 0.490) (Table 4).

**Table 4:** Distribution of demographic and clinical parameters according to pancreatic duct variations

		Pancreatic Duct Variations		
		No	Yes	*p*
Gender	Female	543 (57.3%)	17 (65.4%)	0.413*
	Male	404 (42.7%)	9 (34.6%)	
Acute pancreatitis	No	873 (92.2%)	22 (84.6%)	0.161*
	Yes	74 (7.8%)	4 (15.4%)	
Pancreatic mass and cyst	No	865 (91.3%)	23 (88.5%)	0.490**
	Yes	82 (8.7%)	3 (11.5%)	

*Chi-square test

**Fisher’s exact test; not statistically significant (*p* > 0.05)

In a total of 34 cases, the coexistence of two different biliary tract variations was observed. The most frequent combination was Type IIIa biliary tract variation with distal medial insertion of the cystic duct, observed in seven cases (20.5%). Additionally, pancreatic duct variations were present alongside biliary tract variations in six cases. The distribution of these coexisting variations was equally divided among three groups: Type D biliary variation with pancreas divisum in two cases (33.3%), Type II biliary variation with pancreas divisum in two cases (33.3%), and Type IIIa biliary variation with pancreas divisum in two cases (33.3%).

## Discussion

The biliary system may exhibit a variety of developmental variations at both intrahepatic and extrahepatic levels. These variations are particularly critical during laparoscopic cholecystectomy, as inadvertent ligation can lead to serious complications [12]. The increasing frequency of surgical procedures such as liver resection and transplantation further underscores the importance of a thorough and precise understanding of biliary anatomy. Moreover, these anatomical differences may predispose individuals to gallstone formation, pancreatitis, cholangitis, and biliary malignancies, thereby increasing morbidity [13]. In addition, the duct of Luschka is a clinically important biliary structure that may cause postoperative bile leakage following cholecystectomy. In the present study, it could not be reliably identified on MRCP, likely due to its small caliber, variable anatomy, and the inherent limitations of MRCP in visualizing fine subvesical bile ducts. Nevertheless, awareness of this structure remains essential in surgical practice.

Magnetic resonance cholangiopancreatography (MRCP), which can be performed without contrast agents, has emerged as a rapid and reliable method for evaluating the pancreatic and biliary systems. Assessments using MRCP have revealed various anatomical variations in the pancreas and bile ducts. Intrahepatic biliary variations were observed in 47.8% of cases, while extrahepatic variations were noted in 47.7%. Pancreas divisum was identified in 5.6% of cases and is reported to increase the risk of pancreatitis. Accurate and complete identification of these variations is critical for surgical planning and the prevention of complications [12–14].

In the study conducted by Aljiffry et al. [15], biliary tract variations were observed in 52.3% of 370 patients. The most common variations were reported as Type II and Type IIIa. Similarly, in the study by Jaganathan et al. [12], biliary tract variations were detected in 47.7% of 325 patients evaluated with MRCP, with Type IIIa being the most frequent variation. MRCP examinations performed by Renzulli et al. [16] in 1004 patients revealed intrahepatic and extrahepatic biliary variations ranging from 20% to 55%, with Types IIIa and II being the most common. Getsov and Vladimirov [17] evaluated anatomical variations of the bile ducts in the Bulgarian population using MRCP and reported Type II as the most frequent variation. This study also identified cystic duct variations, with low insertion observed in 24.3%, high insertion in 14.7%, and lateral insertion in 40% of cases.

In our study, biliary tract variations were detected in 522 patients (53.6%). The most frequent variations were Type II (27.78%), Type D (26.25%), and Type IIIa (15.52%). These findings indicate that the variation rates in our cohort are consistent with previously reported literature and support the value of MRCP as a reliable method for identifying biliary tract variations.

In the study by Çoruh et al. [18], biliary tract variations were detected in 42.3% of 274 patients with pancreas divisum identified by MRCP, with Type IIIa being the most frequently observed variation (62.7%). In our study, pancreatic duct variations coexisted with biliary tract variations in six cases; this coexistence was equally distributed among Type D, Type II, and Type IIIa variations (33.3% each). This difference is thought to be due to the evaluation of the general population in our study.

In the study by Bozdağ et al. [19], the impact of extrahepatic biliary tract variations detected by MRCP on the development of acute cholecystitis was investigated, and these variations were reported to be more frequent in patients with cholecystitis. Similarly, Adatepe et al. [20] evaluated biliary tract variations in 1,041 patients and found a higher incidence of cholecystitis in patients with biliary variations. In our study, however, no statistically significant association was observed between biliary tract variations and cholecystitis. This finding may be attributed to the limited sample size, the single-center patient population, and the relatively low incidence of cholecystitis in our cohort.

In the study by Taştemur [21], a significant association was reported between biliary tract variations and the presence of stones, with lateral, medial, and high insertions particularly linked to biliary calculi. Conversely, Khayat et al. [22] evaluated 120 patients and reported that extrahepatic biliary tract variations were not associated with the formation of biliary stones. Renzulli et al. [16], however, reported an association between biliary tract variations and the presence of gallstones in MRCP examinations of 1004 patients. In our study, gallstones in the gallbladder and biliary tracts were observed in 239 cases (49.3%) without biliary tract variations and in 294 cases (60.2%) with biliary tract variations. However, given the retrospective cross-sectional design of the present study, this finding should be interpreted as an association rather than evidence of a causal relationship between biliary tract variations and gallstone formation. Overall, our results are generally consistent with previously reported MRCP-based observational studies.

Pancreatic duct variations were reported in 4.6–9.6% of cases in studies by Çoruh et al. [18], Adıbelli et al. [23], Bülow et al. [24], and Prasad et al. [25], with pancreas divisum being the most common variation. In our study, pancreatic duct variations were identified in 26 cases (2.67%). The lower rate of pancreatic duct variations observed in our study compared to the literature may be attributed to factors such as different geographic and ethnic populations, sample size and selection criteria, and the resolution of MRCP protocols. In addition, variations in diagnostic definitions and the retrospective evaluation of a general clinical population, rather than a selected high-risk cohort, may have contributed to the lower observed prevalence. Nevertheless, the most frequently observed variation in our study was pancreas divisum, consistent with the literature, supporting the general concordance of our findings with previously reported data. Complete visualization of the main pancreatic duct was not achieved in all patients, and partial visualization—most commonly involving the distal duct or side branches—was observed in a subset of cases. This inherent limitation of standard MRCP, particularly in patients without ductal dilatation or overt pancreatic pathology, may have contributed to underestimation of subtle pancreatic duct variations. In addition, secretin-enhanced MRCP, which can improve pancreatic duct visualization by stimulating exocrine secretion, was not used in this study, as it is not part of our routine imaging protocol.

In the study by Adıbelli et al. [23], which included 1158 patients, pancreatic duct variations were reported not to be associated with chronic pancreatitis. Similarly, Bülow et al. [24], in MRCP examinations of 995 volunteers, found no significant relationship between pancreatic duct variations and chronic pancreatitis. Dugic et al. [26] also reported no association between these variations and chronic pancreatitis in a study of 3,234 patients. In our study, acute pancreatitis was observed in 74 cases (7.8%) without pancreatic duct variations and in 4 cases (15.4%) with variations; no statistically significant difference was found between the two groups. While there are no studies in the literature specifically examining the relationship between pancreatic duct variations and acute pancreatitis, chronic pancreatitis often develops from recurrent acute pancreatitis attacks, making our findings partially consistent with existing literature. This difference in outcome definitions (acute versus chronic pancreatitis) may partly explain why our results differ from studies reporting an association between pancreas divisum and pancreatitis.

Kromrey et al. [27] reported that pancreatic cysts were newly identified in 12.9% of cases. Adıbelli et al. [23] detected pancreas divisum in 1,628 MRCP examinations (5.5%) and observed pancreaticobiliary tumors in 7.8% of these patients, whereas the tumor incidence in the general population was 3.5%. Weaver et al. [28] reported that the global incidence of HPB primary malignancies has increased by 1.43%. In our study, pancreatic cysts were observed in 5.5% of cases, pancreatic masses in 3.1%, gallbladder tumors in 0.5%, and biliary tract masses in 0.3%. These rates are lower than those reported in the literature, which may be related to regional differences and the use of contrast-enhanced upper abdominal CT or MRI primarily in patients with suspected tumors.

Despite the high prevalence of biliary anatomical variations, routine preoperative MRCP for all cholecystectomy patients is not warranted. MRCP should be considered selectively, particularly before complex hepatobiliary surgeries or in patients with suspected atypical anatomy, inconclusive conventional imaging, or recurrent biliary symptoms. Currently, no reliable clinical or laboratory criteria exist to predict clinically significant biliary variations; therefore, MRCP primarily serves as a problem-solving tool to clarify anatomy and reduce the risk of bile duct injury in selected cases.

Our study has several limitations. First, the retrospective design may introduce biases during data collection and increase the likelihood of missing or inaccurate data. Second, the selection of patients from a single center, most of whom underwent MRCP for suspected biliary or pancreatic disease, may not fully represent the general population, introducing substantial selection bias and limiting the generalizability of the findings to asymptomatic or broader populations. Third, the sensitivity of MRCP in detecting certain small or rare anatomical variations is limited, which may affect the reported incidence of these variations. The absence of some biliary tract variations in our cohort may be attributed to these factors. In addition, as this study was based on a single MRCP examination, the reported associations between ductal variations and hepatobiliary or pancreatic diseases represent coexistence at the time of imaging rather than temporal or causal relationships, and no longitudinal follow-up was available to assess disease progression. Therefore, information regarding recurrent episodes of acute pancreatitis could not be reliably evaluated. Although the association between biliary tract variations and gallstones remained significant after adjusting for age and sex, the lack of data on metabolic risk factors and comorbidities limits the ability to fully account for potential confounding, and the results should be interpreted cautiously. Finally, the small number of patients with pancreatic duct variations may reduce statistical power when evaluating associations with pancreatitis and contribute to some findings not reaching significance.

## Conclusion

Biliary tract variations were detected in more than half of the patients in our study, with Types II and IIIa being the most frequently observed. Pancreatic duct variations were less common, with pancreas divisum representing the most prevalent type. Gallstones were more frequently observed in patients with biliary tract variations, representing a statistically significant association rather than a causal relationship. No significant differences were observed between groups in terms of acute cholecystitis or acute pancreatitis.

These findings demonstrate that MRCP is a reliable and effective method for detecting variations in the biliary and pancreatic ducts. Considering the clinical significance of these variations can contribute to preventing complications and optimizing treatment planning in diagnostic and surgical procedures. Future studies conducted in larger populations and with prospective designs are warranted to validate these findings and enhance their generalizability.

## Data Availability

No datasets were generated or analysed during the current study.
